# Low circulating arachidonic acid is associated with macroalbuminuria in diabetic patients: a cross-sectional examination of the KAMOGAWA-DM cohort study

**DOI:** 10.1186/s12882-021-02271-8

**Published:** 2021-02-23

**Authors:** Takuro Okamura, Hanako Nakajima, Yoshitaka Hashimoto, Saori Majima, Takafumi Senmaru, Emi Ushigome, Naoko Nakanishi, Masahide Hamaguchi, Mai Asano, Masahiro Yamazaki, Hiroshi Takakuwa, Michiaki Fukui

**Affiliations:** 1 Department of Endocrinology and Metabolism, Kyoto Prefectural University of Medicine, Kyoto, 602-8566 Japan; 2 Agilent Technologies, Chromatography Mass Spectrometry Sales Department, Life Science and Applied Markets Group, Tokyo, 192-8510 Japan

**Keywords:** Cohort study, Epidemiology, Fatty acids, Arachidonic acid, Diabetic nephropathy

## Abstract

**Background:**

Diabetic nephropathy, a major complication of diabetes, is the primary risk factor for dialysis, cardiovascular diseases, and mortality. Dietary fatty acids (FAs) have been revealed to be related with cardiovascular diseases in the general populations. The aim of this study was to investigate the association of circulating FAs with diabetic nephropathy.

**Methods:**

In this cross-sectional study, 190 Japanese patients with type 2 diabetes were included. Circulating FAs were measured by gas chromatography-mass spectrometry. Spearman rank correlation coefficients were used to investigate the association between the logarithm of FAs and the logarithm of urinary albumin excretion (UAE). We have performed logistic regression analysis to determine the effect of FAs on the presence of macroalbuminuria, defined as UAE value ≥300 mg/g creatinine.

**Results:**

Mean age, body mass index, and duration of diabetes were 62.7 ± 12.1 years, 25.0 ± 4.5 kg/m^2^, and 9.8 ± 8.7 years, respectively. In total, 26 patients were diagnosed with macroalbuminuria. The logarithm of circulating arachidonic acid (AA) was negatively associated with the logarithm of UAE (*r* = − 0.221, *p* = 0.002). Additionally, circulating AA in patients with macroalbuminuria was lower than that in patients without macroalbuminuria (112.3 ± 75.3 mg/day vs. 164.8 ± 66.0 mg/day, *p* <  0.001). The logarithm of circulating AA was associated with the presence of macroalbuminuria after adjusting for covariates (odds ratio of Δ1 incremental: 0.32, 95% confidence interval: 0.10–0.99, *p* = 0.042).

**Conclusions:**

Circulating AA was negatively associated with UAE and the presence of macroalbuminuria.

**Supplementary Information:**

The online version contains supplementary material available at 10.1186/s12882-021-02271-8.

## Background

Diabetic nephropathy is a major complication of type 2 diabetes mellitus (T2DM), and is the most common cause of end-stage renal disease. The prevalence of diabetic nephropathy is increasing worldwide [1]. Further, diabetic nephropathy, especially macroalbuminuria, is reported to be the primary risk factor for cardiovascular disease [2], and, eventually, mortality [3]. Thus, the prevention and treatment of diabetic nephropathy, especially macroalbuminuria, are important for the prevention of renal and cardiovascular events and death.

The role of fatty acids (FAs) in inflammation and related chronic diseases is well established. Originally, albumin is an FA transport protein, and hyperfiltration in diabetes causes a high concentration of FAs to enter the tubular space, along with albumin [4]. Circulating FAs are composed of saturated FAs without double bonds, monounsaturated FAs with a single double bond, and polyunsaturated FAs (PUFAs) with multiple double bonds. Several studies have reported that increased influx of these FAs, especially saturated FAs, contributes to the worsening of tubular damage [5, 6]. In addition to saturated FAs, we should focus on the effects of the other types of FAs in organ damage through lipotoxicity. Previous studies reported that n-3 PUFAs, such as eicosapentaenoic acid (EPA) and docosahexaenoic acid, have anti-inflammatory and cardiovascular protective properties [7–9]. On the other hand, n-6 PUFA, such as arachidonic acid (AA), produces a series of lipid mediators called eicosanoids (prostaglandins and leukotrienes), which play a central role in enhancing vascular permeability, and neutrophil infiltration and activation during the early stages of the inflammatory response [10]. In addition, a low ratio of serum EPA to AA has been reported to be a risk factor for cardiovascular events [11–13]. Conversely, several previous studies demonstrated that moderate intake of dietary n-6 PUFAs lowers the risk of coronary artery disease [14] and circulating n-6 PUFAs were inversely associated with total mortality and coronary artery disease mortality [15].

In recent years, it has become clear that the concentration of each circulating FAs in patients with diabetes differed from those of people without diabetes and that these differences may contribute to the development of organ damage [16–18]. However, the association between these circulating FAs, especially detail components of FAs, and diabetic nephropathy has not yet been clarified.

Thus, we conducted the present cross-sectional study with an aim to determine the relation between circulating FAs and diabetic nephropathy.

## Methods

### Study design and participants

The KAMOGAWA-DM cohort study, is an ongoing prospective cohort study that began in 2014 [19], included the outpatient clinics of the Kyoto Prefectural University of Medicine (Kyoto, Japan) and the Kameoka Municipal Hospital (Kameoka, Japan). For the present cross-sectional study, we included T2DM patients in whom FA measurements were performed. We excluded patients taking EPA medication. This study was approved by the hospital’s Ethics Committee. T2DM was diagnosed based on the criteria by the American Diabetes Association [20].

### Data collection

The background factors of patients (i.e., sex, age, disease duration, smoking, and the history of cardiovascular disease (CVD)) was gathered from their medical records. CVD was defined as myocardial infarction and stroke. Blood pressure was measured in an outpatient setting. After an overnight fast, venous blood samples were collected from the patients and fasting plasma glucose, triglycerides, total cholesterol, high-density lipoprotein cholesterol, low-density lipoprotein cholesterol, creatinine, uric acids, and C-peptide were measured. Hemoglobin A1c (HbA1c) levels were measured by high-performance liquid chromatography, ant the values are presented as the National Glycohemoglobin Standardization Program unit. An index of insulin resistance using serum C-peptide concentration, 20/(fasting C-peptide × fasting plasma glucose) [21], was used. All patients submitted spot morning urine samples, and urinary albumin excretion (UAE) was measured by immunoturbidimetric assay. Mean UAE in the present study was used as the average of the three urinary measurements. Macroalbuminuria was defined as UAE > 300 mg/g creatinine [22].

Diabetic neuropathy was diagnosed based on the criteria by The Diagnostic Neuropathy Study Group [23]. Retinopathy was classified into four categories according to the ophthalmologist’s diagnosis as follows: NDR, no diabetic retinopathy; SDR, simple diabetic retinopathy; PPDR, pre-proliferative diabetic retinopathy; and PDR, proliferative diabetic retinopathy.

### Estimation and assessment of the patients’ habitual diet and nutritional intake

A brief-type self-administered diet history questionnaire (BDHQ) was adopted for the assessment of each patient’s habitual diet and nutritional intake [24]. The details of the BDHQ are provided elsewhere [24]. In brief, the BDHQ estimates the dietary intake of 58 food items over the past month from respondents’ memories using a computer algorithm, based on the Standard Tables of Food Composition in Japan [25]. And then, the dietary total energy (kcal/day), carbohydrate (g/day), protein (g/day), total fat (g/day), saturated FAs (g/day), monosaturated FAs (g/day), PUFAs (g/day), n-3 FAs (g/day), n-6 FAs (g/day), total fiber (g/day), cholesterol (g/day), alcohol (g/day), and sodium (g/day) intake were estimated using this calculation program of the BDHQ.

### Measurement of circulating free fatty acids

The composition of FAs in frozen serum samples was measured by GC/MS, Agilent 7890B/5977B (Agilent Technologies, Santa Clara, CA, USA). We metylated 25 μl of serum using a FA methylation kit (Nacalai Tesque, Kyoto, Japan), and loaded the final product onto a Varian capillary column (DB-FATWAX UI; Agilent Technologies). The capillary column used for FA separation was CP-Sil 88 for FAME (100 m × an inner diameter of 0.25 mm × membrane thickness of 0.20 μm, Agilent Technologies). We set the temperature in column at 100 °C for 4 min and then increased gradually by 3 °C/min to 240 °C and held there for 7 min. We injected the samples in split mode at a split ratio of 5:1. Each FA methyl ester was detected in the selected ion monitoring mode. All results were normalized to the peak height of the C17:0 internal standard [26].

### Statistical analysis

JMP (ver. 13.0) software (SAS Institute, Cary, NC, USA) was used for all analyses of the patients’ data. Probability values of < 0.05 were accepted as significant. Categorical variables were expressed as numbers, and continuous variables were presented as the mean ± SD. To assess the statistical significance of the differences between groups, the chi-square test for categorical variables and the Wilcoxon signed-rank test for continuous variables was used because the continuous variables did not follow a normal distribution. Spearman rank correlation coefficients were used to investigate the association between the logarithm of FAs and the logarithm of UAE. Then, because AA was found to be significantly correlated with the logarithm UAE, the odds ratio (OR) and 95% confidence interval (CI) of AA and other variables in the presence of macroalbuminuria were calculated by performing univariate and multivariate logistic regression analyses. We adjusted for age and sex (Model 2) and further adjusted for body mass index (BMI), disease duration, index of insulin resistance, total cholesterol, systolic blood pressure, smoking status, physical activity, total energy, usage of renin-angiotensin system inhibitors, creatinine and the history of CVD (Model 3). In addition, the area under the curve (AUC) of circulating arachidonic acid concentration for the presence of macroalbuminuria was calculated by the receiver operating characteristic (ROC) curve.

## Results

**Fig. 1:**
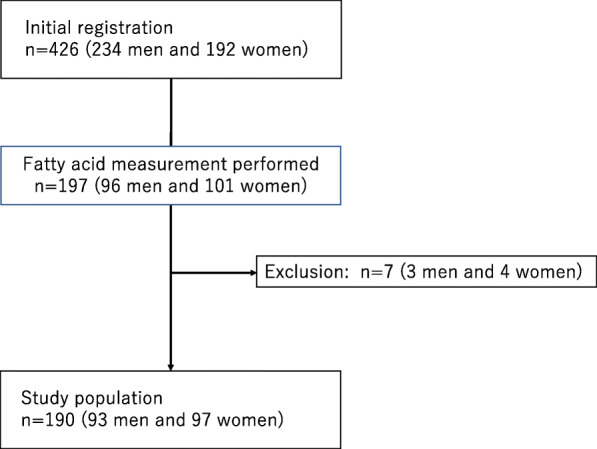
The registration of patients

In this study, the BDHQ was administered to 426 patients (234 men and 192 women) with T2DM. Among them, we measured circulating FAs in 197 patients (96 men and 101 women) using the GC/MS system. Moreover, we excluded 7 patients with EPA medication usage (Fig. 1).

The clinical characteristics of the 190 patients with T2DM are summarized in Table 1. Mean age, HbA1c, and UAE were 62.7 ± 12.1 years, 7.5 ± 1.6%, and 202.0 ± 583.0 mg/gCr, respectively. The number (percentage) of patients with macroalbuminuria was 27 (13.7%).

**Table 1:** Clinical characteristics of the study patients

	Total (***n*** = 190)
Sex, men/women	93/97
Age, yrs	65.0 (8.0)
Duration of diabetes, yrs	7.0 (11.0)
BMI, kg/m^2^	24.4 (5.1)
Systolic blood pressure, mmHg	133.0 (22.0)
Diastolic blood pressure, mmHg	81.0 (15.0)
Fasting plasma glucose, mmol/L	6.9 (2.2)
Hemoglobin A1c, %	7.1 (1.7)
C-peptide, nmol/L	0.5 (0.4)
20/C-peptide/fasting plasma glucose	5.9 (4.2)
Triglycerides, mmol/L	1.3 (0.6)
Total cholesterol, mmol/L	5.2 (1.1)
High-density lipoprotein cholesterol, mmol/L	1.5 (0.5)
Low-density lipoprotein cholesterol, mmol/L	3.0 (0.9)
Creatinine, μmol/L	66.7 (26.6)
Estimated GFR, mL/min/1.73m^2^	71.7 (22.6)
Uric acids, mmol/L	306.3 (107.1)
Urine albumin to creatinine ratio, mg/gCr	20.4 (66.6)
Diabetic nephropathy	115/49/26
Normoalbuminuria	115 (60.5)
Microalbuminuria	49 (25.8)
Macroalbuminuria	26 (13.7)
Diabetic retinopathy, NDR/SDR/PPDR/PDR	163/13/4/10
Diabetic neuropathy −/+	150/40
Never smoker	120 (63.2)
Ex-smoker	39 (20.5)
Current smoker	31 (16.3)
Usage of RAS inhibitors	70 (36.8)
Energy intake/day, kcal	1657.4 (749.0)
Carbohydrate intake, g/day	200.8 (90.1)
Protein intake, g/day	66.8 (36.2)
Total fat intake, g/day	51.6 (28.4)
Saturated fatty acids intake, g/day	13.1 (8.2)
Monosaturated fatty acids intake, g/day	18.7 (10.3)
Polyunsaturated fatty acids intake, g/day	12.7 (5.3)
n-3 fatty acids intake, g/day	2.8 (1.5)
n-6 fatty acids intake, g/day	9.7 (5.7)
Total fiber intake, g/day	11.5 (7.0)
Cholesterol intake, g/day	388.0 (269.0)
Alcohol intake, g/day	0.0 (3.4)
Sodium intake, g/day	10.7 (5.3)

Data are expressed as number (%) of subjects or median (IQR)

*BMI* body mass index, *GFR* glomerular filtration rate, *IQR* interquartile range, *NDR* no diabetic retinopathy, *PDR* proliferative diabetic retinopathy, *PPDR* pre- proliferative diabetic retinopathy, *SDR* simple diabetic retinopathy

Mean circulating FA concentrations are shown in Table 2. Pentadecanoic acid (C15:0), oleic acid (C18:1(9c)), C18:3(9c12t15t) + C18:3(9c12c15t), α-Linolenic acid (C18:3(9c12c15c)), eicosenoic acid (C20:1), eicosadienoic acid (C20:2), AA (C20:4n6), heneicosylic acid (C21:0), behenic acid (C22:0), and tricosanoic acid (C23:0) were significantly lower in patients with macroalbuminuria than in those without macroalbuminuria.

**Table 2:** Circulating fatty acids of the study patients

Fatty acids, μg/ml	All (***n*** = 190)	Normo−/ Micro-albuminuria (***n*** = 164)	Macro-albuminuria (***n*** = 26)	***p***-value
Caproic acid (C6:0)	0.5 (2.3)	0.5 (2.4)	0.6 (1.9)	0.808
Caprylic acid (C8:0)	0.5 (2.8)	0.5 (2.6)	1.1 (4.0)	0.245
Capric acid (C10:0)	1.0 (2.9)	0.9 (2.7)	1.4 (4.1)	0.383
Undecyl acid (C11:0)	1.4 (1.9)	1.4 (1.8)	1.3 (2.3)	0.662
Lauric acid (C12:0)	5.4 (8.0)	5.3 (7.5)	6.1 (11.0)	0.640
Tridecyl acid (C13:0)	2.5 (2.5)	2.6 (2.5)	2.0 (2.8)	0.230
Myristic acid (C14:0)	27.4 (16.8)	27.6 16.9)	25.8 (16.4)	0.603
Myristoleic acid (C14:1(9c))	4.9 (5.5)	5.1 (5.6)	3.7 (4.5)	0.226
Pentadecanoic acid (C15:0)	6.0 (2.7)	6.2 (2.6)	4.8 (2.9)	0.011
Pentadecenoic acid (C15:1)	6.0 (2.7)	2.3 (28.5)	0.0 (0.0)	0.691
Palmitic acid (C16:0)	743.5 (314.8)	747.9 (315.9)	715.4 (312.4)	0.626
Palmitoleic acid (C16:1(9c))	47.6 (37.2)	46.3 (34.3)	55.2 (51.9)	0.259
Stearic acid (C18:0)	212.7 (84.4)	216.2 (86.1)	190.3 (70.2)	0.147
Petroselinic acid (C18:1(6c))	821.7 (438.0)	815.8 (435.6)	858.6 (460.4)	0.645
Oleic acid (C18:1(9c))	1009.0 (853.4)	1052.2 (886.2)	736.7 (543.4)	0.040
Vaccenic acid (C18:1(11 t))	27.5 (150.3)	31.9 (161.4)	0.0 (0.0)	0.316
(C18:1(11c))	57.3 (33.0)	58.1 (31.7)	52.6 (40.8)	0.431
Linolelaidic acid (C18:2(9t12c))	80.9 (257.0)	86.5 (268.8)	45.9 (164.0)	0.455
Linoleic acid (C18:2(9c12c))	868.1 (370.9)	878.8 (381.4)	800.5 (293.6)	0.319
C18:3(9t12t15c) + C18:3(9t12c15t)	7.1 (9.4)	7.6 (9.6)	3.9 (7.5)	0.032
ɤ- Linolenic acid (C18:3n6)	5.2 (4.7)	5.5 (4.6)	3.5 (5.2)	0.021
C18:3(9c12t15t) + C18:3(9c12c15t)	9.5 (7.4)	10.0 (7.2)	6.3 (7.9)	0.009
C18:3(9c12t15c)	0.9 (2.4)	0.8 (2.3)	1.5 (2.6)	0.149
C18:3(9t12c15c)	1.9 (7.0)	2.1 (7.5)	0.5 (2.4)	0.276
α-Linolenic acid (C18:3(9c12c15c))	22.2 (10.4)	22.8 (10.5)	18.8 (8.7)	0.034
Arachidic acid (C20:0)	0.4 (2.2)	0.5 (2.4)	0.0 (0.0)	0.330
Eicosenoic acid (C20:1)	6.7 (3.8)	7.1 (3.5)	4.3 (4.3)	< 0.001
Eicosadienoic acid (C20:2)	9.5 (4.3)	9.9 (4.2)	7.5 (4.1)	0.007
Eicosatrienoic acid (C20:3 (11,14,17))	0.1 (0.9)	0.1 (0.9)	0.0 (0.0)	0.692
Dihomo-gamma-linolenic acid (C20:3n6)	27.6 (10.7)	28.0 (10.3)	24.9 (12.9)	0.176
Arachidonic acid (C20:4n6)	157.6 (69.6)	164.8 (66.0)	112.3 (75.3)	< 0.001
Eicosapentaenoic acid (C20:5n3)	42.0 (24.5)	41.1 (27.1)	47.5 (29.8)	0.271
Heneicosylic acid (C21:0)	4.5 (4.7)	5.1 (4.7)	1.2 (3.1)	< 0.001
Behenic acid (C22:0)	13.2 (8.6)	14.0 (8.3)	8.7 (9.2)	0.004
Erucic acid (C22:1n9)	1.9 (4.5)	2.0 (4.6)	1.3 (3.6)	0.432
Docosadienoic acid (C22:2)	5.9 (5.9)	6.1 (6.0)	5.1 (4.7)	0.455
Docosahexaenoic acid (C22:6n3)	146.1 (63.9)	147.1 (64.1)	140.0 (63.7)	0.593
Tricosanoic acid (C23:0)	16.1 (7.2)	16.8 (6.8)	11.5 (7.7)	< 0.001
Lignoceric acid (C24:0)	13.0 (13.3)	13.7 (13.4)	8.6 (11.9)	0.033
Nervonic acid (C24:1)	3.4 (6.0)	3.7 (6.2)	1.9 (3.6)	0.159

Data are expressed as mean (SD)

In addition, the mean dietary intakes of nutrients, including FAs, are shown in Table 3. Energy intake in patients with macroalbuminuria tended to be higher than that in patients without macroalbuminuria (1891.5 ± 809.9 kcal/day vs. 1649.6 ± 551.3 kcal/day, *p* = 0.069), whereas total fat intake was not significantly different (57.9 ± 22.0 g/day vs. 53.0 ± 20.0 g/day, *p* = 0.277). The dietary intake of eicosenoic acid (C20:1), AA (C20:4n6), EPA (C20:5n3), erucic acid (C22:1n9), erucic acid (C22:1n9), docosahexaenoic acid (C22:6n3), and nervonic acid (C24:1) in patients with macroalbuminuria was significantly higher than that in patients without macroalbuminuria.

**Table 3:** Dietary fatty acids intake of the study patients

	All (***n*** = 190)	Normo−/ Micro-albuminuria (***n*** = 164)	Macro-albuminuria (***n*** = 26)	***p***-value
Total energy intake, kcal/day	1682.5 (587.3)	1649.6 (551.3)	1891.5 (809.9)	0.069
Carbohydrate intake, g/day	209.9 (80.6)	204.8 (70.8)	233.3 (129.2)	0.117
Protein intake, g/day	71.7 (28.6)	70.5 (27.4)	81.6 (37.4)	0.090
Total fat, g/day	53.5 (20.0)	53.0 (20.0)	57.9 (22.0)	0.277
Total fiber intake, g/day	12.0 (5.1)	12.0 (4.9)	12.7 (7.0)	0.546
Saturated fatty acid intake, g/day	13.7 (5.7)	13.6 (5.8)	14.2 (5.4)	0.666
Monosaturated fatty acids intake, g/day	19.3 (7.5)	19.1 (7.5)	21.3 (8.4)	0.192
Polyunsaturated fatty acids intake, g/day	13.2 (4.9)	13.1 (4.9)	14.4 (5.8)	0.273
Caproic acid (C6:0)	92.3 (83.2)	95.0 (85.4)	75.0 (66.0)	0.284
Caprylic acid (C8:0)	90.8 (85.6)	94.7 (89.4)	65.1 (48.7)	0.123
Capric acid (C10:0)	151.5 (128.2)	156.6 (132.4)	118.0 (92.0)	0.179
Lauric acid (C12:0)	360.9 (358.9)	377.3 (376.6)	253.2 (178.9)	0.123
Myristic acid (C14:0)	1040.4 (563.6)	1039.6 (572.2)	1045.4 (512.8)	0.963
Myristoleic acid (C14:1(9c))	77.8 (43.6)	77.2 (44.2)	81.9 (39.9)	0.627
Pentadecanoic acid (C15:0)	99.4 (52.4)	98.6 (52.7)	104.7 (50.9)	0.605
Palmitic acid (C16:0)	8234.1 (3281.5)	8159.4 (3291.8)	8724.8 (3241.2)	0.443
Palmitoleic acid (C16:1(9c))	873.4 (394.2)	847.8 (382.3)	1041.7 (437.5)	0.028
Stearic acid (C18:0)	3007.0 (1250.8)	2981.8 (1255.6)	3172.2 (1233.5)	0.498
Oleic acid (C18:1(9c))	17,282.8 (6881.2)	17,066.6 (6782.7)	18,701.6 (7503.6)	0.290
Linoleic acid (C18:2(9c12c))	9975.4 (3823.8)	9898.3 (3745.0)	10,481.8 (4364.0)	0.497
ɤ- Linolenic acid (C18:3n6)	7.5 (5.1)	7.4 (5.0)	8.1 (5.8)	0.573
α-Linolenic acid (C18:3(9c12c15c))	1634.0 (684.6)	1616.7 (664.1)	1747.6 (814.3)	0.395
Arachidic acid (C20:0)	154.1 (60.3)	152.5 (58.9)	165.1 (69.5)	0.350
Eicosenoic acid (C20:1)	564.5 (302.7)	540.9 (279.5)	718.8 (398.8)	0.008
Eicosadienoic acid (C20:2)	49.3 (21.9)	48.2 (21.6)	56.1 (23.2)	0.107
Dihomo-gamma-linolenic acid (C20:3n6)	31.9 (14.0)	31.6 (13.8)	37.0 (14.6)	0.063
Arachidonic acid (C20:4n6)	188.9 (82.1)	183.6 (80.3)	223.3 (87.8)	0.031
Eicosapentaenoic acid (C20:5n3)	402.9 (277.5)	385.0 (267.2)	520.1 (319.9)	0.029
Behenic acid (C22:0)	79.7 (32.6)	79.0 (31.7)	83.8 (38.4)	0.512
Erucic acid (C22:1n9)	406.3 (309.9)	383.7 (24.9)	548.6 (62.4)	0.015
Docosahexaenoic acid (C22:6n3)	664.6 (422.8)	636.6 (404.4)	848.9 (499.7)	0.025
Lignoceric acid (C24:0)	32.6 (14.1)	32.1 (13.8)	35.5 (16.4)	0.280
Nervonic acid (C24:1)	59.8 (36.4)	57.1 (34.4)	77.9 (44.2)	0.010

Data are expressed as mean (SD)

Moreover, we investigated the association between log circulating FAs and log UAE. Logarithm AA showed a significant correlation (*r* = − 0.221, *p* = 0.002) (Table 4). Therefore, we performed logistic regression analyses to investigate the association between logarithm AA and the presence of macroalbuminuria. In univariate logistic regression analysis, logarithm AA was negatively associated with the presence of macroalbuminuria (Model 1, OR of Δ1 incremental: 0.03, 95% CI: 0.01–0.21, *p* <  0.001). Multivariate logistic regression analysis revealed that logarithm AA was negatively associated with the presence of macroalbuminuria, even after adjusting for covariates (OR of Δ1 incremental: 0.32, 95% CI: 0.10–0.99, *p* = 0.042) (Table 5).

**Table 4:** Correlation coefficient between logarithm urinary albumin excretion and serum fatty acids

Fatty acids, μg/ml	***r***	***p***-value
Caproic acid (C6:0)	0.042	0.563
Caprylic acid (C8:0)	0.114	0.118
Capric acid (C10:0)	−0.044	0.546
Undecyl acid (C11:0)	−0.018	0.802
Lauric acid (C12:0)	0.043	0.553
Tridecyl acid (C13:0)	−0.026	0.727
Myristic acid (C14:0)	0.011	0.882
Myristoleic acid (C14:1(9c))	0.105	0.148
Pentadecanoic acid (C15:0)	−0.200	0.007
Pentadecenoic acid (C15:1)	−0.030	0.675
Palmitic acid (C16:0)	−0.010	0.850
Palmitoleic acid (C16:1(9c))	0.029	0.688
Stearic acid (C18:0)	−0.070	0.308
Petroselinic acid (C18:1(6c))	0.022	0.766
Oleic acid (C18:1(9c))	−0.081	0.268
Vaccenic acid (C18:1(11 t))	−0.051	0.487
(C18:1(11c))	−0.061	0.406
Linolelaidic acid (C18:2(9t12c))	−0.038	0.606
Linoleic acid (C18:2(9c12c))	−0.072	0.321
C18:3(9t12t15c) + C18:3(9t12c15t)	−0.129	0.075
ɤ- Linolenic acid (C18:3n6)	−0.089	0.220
C18:3(9c12t15t) + C18:3(9c12c15t)	−0.132	0.069
C18:3(9c12t15c)	0.100	0.169
C18:3(9t12c15c)	−0.029	0.688
α-Linolenic acid (C18:3(9c12c15c))	−0.135	0.064
Arachidic acid (C20:0)	−0.061	0.402
Eicosenoic acid (C20:1)	−0.002	0.977
Eicosadienoic acid (C20:2)	−0.175	0.016
Eicosatrienoic acid (C20:3 (11,14,17))	0.084	0.249
Dihomo-gamma-linolenic acid (C20:3n6)	−0.100	0.171
Arachidonic acid (C20:4n6)	−0.221	0.002
Eicosapentaenoic acid (C20:5n3)	0.065	0.376
Heneicosylic acid (C21:0)	0.021	0.776
Behenic acid (C22:0)	−0.183	0.011
Erucic acid (C22:1n9)	−0.014	0.847
Docosadienoic acid (C22:2)	−0.020	0.787
Docosahexaenoic acid (C22:6n3)	−0.027	0.709
Tricosanoic acid (C23:0)	−0.195	0.007
Lignoceric acid (C24:0)	−0.149	0.040
Nervonic acid (C24:1)	−0.062	0.396

**Table 5:** Logistic regression analyses for macroalbuminuria

	Model 1		Model 2		Model 3	
	Odds ratio (95% CI)	*p*-value	Odds ratio (95% CI)	*p*-value	Odds ratio (95% CI)	*p*-value
Age, yrs	1.07 (1.03–1.13)	< 0.001	1.06 (1.01–1.12)	0.015	1.03 (0.94–1.13)	0.509
Men	4.43 (1.70–11.5)	< 0.001	3.38 (1.23–9.32)	0.019	4.73 (0.47–47.5)	0.186
Disease duration, yrs	1.06 (1.01–1.10)	0.010	–	–	1.08 (0.96–1.22)	0.180
Body mass index, kg/m^2^	0.99 (0.91–1.09)	0.878	–	–	1.05 (0.83–1.32)	0.684
20/(fasting C-peptide × fasting plasma glucose)	0.99 (0.93–1.06)	0.596	–	–	1.03 (0.82–1.29)	0.790
Creatinine, μmol/L	1.07 (1.04–1.09)	< 0.001	–	–	1.07 (1.02–1.15)	< 0.001
Total cholesterol, mmol/L	0.81 (0.54–1.21)	0.311			1.05 (0.40–2.74)	0.921
Systolic blood pressure, mmHg	1.03 (1.01–1.05)	0.003	–	–	1.02 (0.96–1.08)	0.590
Never smoker	1.00 (Reference)	–	–	–	1.00 (Reference)	–
Ex-smoker	2.90 (1.02–8.22)	0.045	–	–	0.96 (0.11–8.13)	0.974
Current smoker	3.01 (1.14–7.91)	0.026	–	–	2.54 (0.31–20.6)	0.381
Exercise	0.76 (0.33–1.76)	0.527	–	–	0.38 (0.07–1.96)	0.248
Total Energy, kcal/day	1.00 (0.99–1.00)	0.095	–	–	0.99 (0.99–1.00)	0.431
RAS inhibitors	4.85 (1.98–11.87)	< 0.001	–	–	5.04 (0.92–27.6)	0.062
History of CVD	7.57 (2.02–28.4)	0.004			1.52 (0.02–99.7)	0.844
Log arachidonic acid	0.03 (0.01–0.21)	< 0.001	0.46 (0.24–0.87)	0.016	0.32 (0.10–0.99)	0.042

Model 1 was univariate; Model 2: adjusted for age, sex; Model 3: model 2 plus body mass index, disease duration, 20/(fasting C-peptide × fasting plasma glucose), uric acids, total cholesterol, systolic blood pressure, smoking status, physical activity, total energy, RAS inhibitors usage, and history of CVD

*CVD* cardiovascular disease, *RAS* renin-angiotensin system, *UAE* urinary albumin excretion

**Fig. 2:**
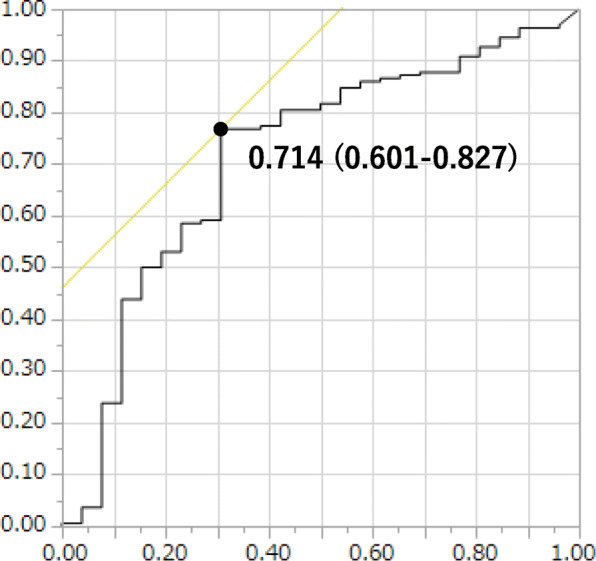
Area under the receiver operating characteristic (ROC) curve (AUC) [95% confidence interval (CI)] of several factors for the presence of macroalbuminuria. AUC of circulating arachidonic acid concentration was 0.714 (95% CI 0.601–0.827), and the cut-off level of serum AAs with diabetic patients for predicting the presence of macroalbuminuria was 327.5 μg/ml

Additionally, in ROC analyses, AUC of circulating arachidonic acid concentration was 0.714 (95% CI 0.601–0.827) and the cut-off level of serum AAs with diabetic patients for predicting the presence of macroalbuminuria was 327.5 μg/ml (Fig. 2).

## Discussion

In the present study, in a cohort of 190 Japanese individuals with T2DM, we investigated the association between circulating FAs and diabetic nephropathy, and we revealed that circulating AA is negatively associated with UAE and the presence of macroalbuminuria.

Possible explanations for the association between circulating AA and diabetic nephropathy are described below. AA is a component of biological membranes and is involved in the generation of prostaglandins, which are the most representative physiologically active lipids, through cyclooxygenase (COX). Prostacyclin (PGI2), a type of prostaglandin, has vasodilatory effects [27], antiplatelet effects [28], angiogenesis [29], and protective effects on vascular endothelium [30]. PGI2 is produced from prostaglandin H2 (PGH2) by prostacyclin synthase [31]. In addition, prostaglandin E1 has a protective effect on diabetic kidney disease by decreasing UAE [32], whereas prostaglandin E2 decrease myocardium contractility and cause cardiorenal syndrome [33]. AA is associated with PGH2, through separation from phospholipids in the cell membrane and receiving COX. PGI2 is well known for its regulation of renal hemodynamics, tubular transport, and renin release and plays an important role by coupling with its receptors and the downstream signals in various types of renal diseases including chronic kidney disease [34]. Therefore, circulating AA levels in patients with macroalbuminuria were significantly lower than those with normoalbuminuria and microalbuminuria, suggesting that the body promoted the production of PGI2 to prevent progression of nephropathy and the other CVD.

On the contrary, AA has been reported to potentiate hypoxia-induced vascular endothelial growth factor (VEGF) expression through the Notch-1, Wnt-1, and HIF-1alpha pathways [35]. In several previous studies, VEGF was proven to have an ameliorating effect on diabetic nephropathy [36], normalization of hyperpermeability in the glomeruli of diabetic nephropathy [37], and in restoring endothelial glycocalyx in diabetic nephropathy [38]. It is suggested that AA is consumed for PGI2 production and the concentration of AA in the body is lowered, which may reduce the effects of VEGF from being fully activated. Collectively, patients with advanced diabetic nephropathy might consume more AA to raise levels of PGI2, which leads to lower circulating AA levels and decreased AA reduce the effect of VEGF and induces further progression of nephropathy. This is potentially the reason why circulating AA levels in patients with diabetic nephropathy are significantly lower than those of patients without this condition, although dietary AA intake in patients with diabetic nephropathy is higher than that in patients without diabetic nephropathy.

There are three major limitations in this study that could be addressed in future research. First, this study had a cross-sectional design; thus, further studies are required to establish the causal relationship between circulating AA levels and diabetic nephropathy. Second, the sample size in this study was relatively small. Thus, further large-scale studies are needed. Third, we did not check circulating prostaglandin level.

## Conclusions

In conclusion, this study provides the first demonstration that circulating AA levels are negatively associated with UAE and that these levels are significantly lower in patients with macroalbuminuria than in those without macroalbuminuria. Future prospective studies on the relationship between circulating AA levels and the incidence of diabetic nephropathy and the differences in mechanism between dietary AA and circulating AA are required.

## Supplementary Information

**Additional file 1:.**

**Additional file 2:.**

## Data Availability

The datasets used and/or analysed during the current study are available from the corresponding author on reasonable request.

## Abbreviations

AA: Arachidonic acid
BDHQ: Brief-type self-administered diet history questionnaire
BMI: Body mass index
CI: Confidence interval
COX: Cyclooxygenase
EPA: Eicosapentaenoic acid
FA: Fatty acid
HbA1c: Hemoglobin A1c
PGH2: Prostaglandin H2
PGI2: Prostacyclin
PUFA: Polyunsaturated fatty acid
T2DM: Type 2 diabetes mellitus
UAE: Urinary albumin excretion
VEGF: Vascular endothelial growth factor
