# Supplementary material for: Low circulating arachidonic acid is associated with macroalbuminuria in diabetic patients: a cross-sectional examination of the KAMOGAWA-DM cohort study
Source: BMC Nephrol. 2021 Feb 23;22:68. doi: 10.1186/s12882-021-02271-8 (PMC7903748; doi:10.1186/s12882-021-02271-8)

同意書

実施責任者
　所属・職　　京都府立医科大学　内分泌・代謝内科学　教授
　　氏　　名　　福井　道明　様

　私（氏名）＿＿＿＿＿＿は、（課題名）　糖尿病患者におけるコホート調査研究　の実施について
（説明者）＿＿＿＿＿より（日時）西暦＿＿＿＿年＿＿月＿＿日、内科外来において説明文書を用いて説明を受け、実施計画の意義、目的、方法、個人情報の保護方法などについて十分理解しましたので計画に参加することを同意いたします。

１　説明を受け（説明文書を参照し）理解した項目（☐の中にご自分でレを付けてください）

☐　計画の意義及び目的　　☐　計画の実施方法
☐　計画への参加は任意であること
☐　参加に同意した場合でも、随時文書により撤回できること
☐　研究対象者等の選定方法
☐　予想される結果、危険性等
☐　研究対象者等への説明及びインフォームド・コンセントの取得方法
☐　個人情報の保護方法
☐　費用負担に関する事項　　☐　試料・資料の保管について

２　研究協力への同意
(1)提供する試料・情報が本研究に使用されることに同意します。
☐　はい　　☐　いいえ
※質問（１）に対して「はい」に✓をつけた方は質問（２）に進み、どちらかに✓をつけてください。

(2)提供する試料・情報が本研究に使用されるとともに、長期間保存され、かつ将来、本学医学倫理審査委員会の承認を受けた上で、新たに計画・実施される研究に使用されることに同意します。
※「いいえ」の場合、本研究終了後、速やかに試料・資料は廃棄します。
☐　はい　　☐　いいえ

西暦＿＿＿＿年＿＿月＿＿日

氏　　名　　印

生年月日
住　　所

電話番号

**施設名**	
**診察券の患者番号**	
**研究ID**	

施設名	
研究ID	
記載日	西暦　______年____月____日
生年月日	西暦　______年____月____日
性別	☐ 男　☐ 女
郵便番号	__________ ー __________
糖尿病と言われたのは何歳ですか	_____歳ごろ　☐　今回初めて
現在の身長、体重を教えてください	身長　________cm、　体重　________kg
20歳時の体重、また最高体重とその時の年齢を教えてください	20歳時の体重_______kg、<br>最高体重________kg、その時の年齢______歳

●御家族のことについて教えてください

血縁者で糖尿病の方はいますか	☐　いない　☐　父　☐　母　☐　両親以外の血縁者

●ライフスタイルについて、ご自身にあてはまるものにレ点をつけて下さい

タバコを吸いますか	☐　現在吸っている　☐　過去に吸っていた<br>☐　吸ったことがない
タバコを吸われている場合、どのくらい喫煙していますか<br>タバコを吸われていた場合、どのくらい喫煙していましたか	1日______本を______年間
飲酒しますか	☐　ほぼ毎日　☐　週3-4回　☐　週1-2回　☐　週１回未満<br>☐　しない
飲酒すると赤くなりますか	☐　はい　☐　いいえ　☐　わからない
同居人はいますか	☐　なし<br>☐　あり　（　☐　親　☐　夫or妻　☐　子　☐　孫<br>☐　その他（　　　　）<br>☐　ペット（　　　　））
結婚について教えてください	☐　現在結婚している　☐　過去に結婚したことがある<br>☐　結婚したことが無い　☐　死別
最終学歴を教えてください	☐　小学校　☐　中学校　☐　高校　☐　高専<br>☐　専門学校　☐　短期大学　☐　大学　☐　大学院

●食事について、当てはまるものにレ点、もしくは記載してください

食事のおおよその時間を教えてください<br>（２４時間表記でご記載ください）	朝　______時頃、　昼　______時頃、　夜　______時頃
朝食を毎日欠かさず食べますか	☐　はい　☐　いいえ
夕食で外食であることはありますか	☐　ほぼ毎日　☐　週3-4回　☐　週1-2回　☐　とらない
夜食をとりますか（夕食以降）	☐　1日2回以上　☐　ほぼ毎日　☐　週3-4回　☐　週1-2回<br>☐　とらない
間食をとりますか	☐　1日2回以上　☐　ほぼ毎日　☐　週3-4回　☐　週1-2回<br>☐　とらない
夜食・間食の時間は何時頃ですか<br>（２４時間表記でご記載ください）	______時頃、　______時頃、　______時頃
朝、昼、夕食で一番量が多いのはどれですか	☐　朝食　☐　昼食　☐　夕食　☐　3食とも同じ

あなたは、この１か月のあいだ、以下の食べ物をどのくらいの頻度で食べていましたか？
もっともあてはまる回答をひとつ選んで、∨ を記入してください。

枠線の中にある3点を結んでください。

良い例

悪い例

コップ1杯くらいの牛乳・ヨーグルト1人前		鶏肉（挽き肉を含む）	豚肉・牛肉・羊肉（挽き肉を含む）	ハム・ソーセージ・ベーコンなどの加工肉	レバー
低脂肪	普通・高脂肪				
毎日2回以上	毎日2回以上	毎日2回以上	毎日2回以上	毎日2回以上	毎日2回以上
毎日1回	毎日1回	毎日1回	毎日1回	毎日1回	毎日1回
週4～6回	週4～6回	週4～6回	週4～6回	週4～6回	週4～6回
週2～3回	週2～3回	週2～3回	週2～3回	週2～3回	週2～3回
週1回	週1回	週1回	週1回	週1回	週1回
週1回未満	週1回未満	週1回未満	週1回未満	週1回未満	週1回未満
飲まなかった	飲まなかった	食べなかった	食べなかった	食べなかった	食べなかった

飲まなかったり、食べなかった場合は、ここに ∨ を記入してください

いか・たこ・えび・貝	骨ごと食べる魚	ツナ缶（まぐろの油漬け）	魚の干物・塩蔵魚・魚介練り製品（塩さば・塩鮭・あじの干物・ちくわ・かまぼこなど）	脂が乗った魚（いわし・さば・さんま・ぶり・にしん・うなぎ・まぐろトロなど）	脂が少なめの魚（さけ・ます・白身の魚・淡水魚・かつおなど）	たまご（鶏の卵1個程度）
毎日2回以上	毎日2回以上	毎日2回以上	毎日2回以上	毎日2回以上	毎日2回以上	毎日2回以上
毎日1回	毎日1回	毎日1回	毎日1回	毎日1回	毎日1回	毎日1回
週4～6回	週4～6回	週4～6回	週4～6回	週4～6回	週4～6回	週4～6回
週2～3回	週2～3回	週2～3回	週2～3回	週2～3回	週2～3回	週2～3回
週1回	週1回	週1回	週1回	週1回	週1回	週1回
週1回未満	週1回未満	週1回未満	週1回未満	週1回未満	週1回未満	週1回未満
食べなかった	食べなかった	食べなかった	食べなかった	食べなかった	食べなかった	食べなかった

とうふ・厚揚げ・豆乳	納豆	いも（すべての種類）	漬け物		生（サラダ）	トマト・トマトケチャップ・トマト煮込み・トマトシチュー
			緑の濃い葉野菜	その他すべて（梅干は除く）	レタス・キャベツ千切りなど（トマトは除く）	
毎日2回以上	毎日2回以上	毎日2回以上	毎日2回以上	毎日2回以上	毎日2回以上	毎日2回以上
毎日1回	毎日1回	毎日1回	毎日1回	毎日1回	毎日1回	毎日1回
週4～6回	週4～6回	週4～6回	週4～6回	週4～6回	週4～6回	週4～6回
週2～3回	週2～3回	週2～3回	週2～3回	週2～3回	週2～3回	週2～3回
週1回	週1回	週1回	週1回	週1回	週1回	週1回
週1回未満	週1回未満	週1回未満	週1回未満	週1回未満	週1回未満	週1回未満
食べなかった	食べなかった	食べなかった	食べなかった	食べなかった	食べなかった	食べなかった

料理に使った野菜（漬け物・サラダ以外）					きのこ（すべての種類）	海草（すべての種類）（だし用は除く）
緑の濃い葉野菜（ブロッコリー・ゴーヤーを含む）	キャベツ・白菜	にんじん・かぼちゃ	だいこん・かぶ	その他の根菜すべて（たまねぎ・ごぼう・れんこんなど）		
毎日2回以上	毎日2回以上	毎日2回以上	毎日2回以上	毎日2回以上	毎日2回以上	毎日2回以上
毎日1回	毎日1回	毎日1回	毎日1回	毎日1回	毎日1回	毎日1回
週4～6回	週4～6回	週4～6回	週4～6回	週4～6回	週4～6回	週4～6回
週2～3回	週2～3回	週2～3回	週2～3回	週2～3回	週2～3回	週2～3回
週1回	週1回	週1回	週1回	週1回	週1回	週1回
週1回未満	週1回未満	週1回未満	週1回未満	週1回未満	週1回未満	週1回未満
食べなかった	食べなかった	食べなかった	食べなかった	食べなかった	食べなかった	食べなかった

洋菓子・クッキー・ビスケット	和菓子	せんべい・スナック菓子・もち・お好み焼きなど	アイスクリーム	みかんなどの柑橘（かんきつ）類	かき・いちご・キウイ	その他のすべての果物（りんご・バナナなど）
□ 毎日2回以上	□ 毎日2回以上	□ 毎日2回以上	□ 毎日2回以上	□ 毎日2回以上	□ 毎日2回以上	□ 毎日2回以上
□ 毎日1回	□ 毎日1回	□ 毎日1回	□ 毎日1回	□ 毎日1回	□ 毎日1回	□ 毎日1回
□ 週4～6回	□ 週4～6回	□ 週4～6回	□ 週4～6回	□ 週4～6回	□ 週4～6回	□ 週4～6回
□ 週2～3回	□ 週2～3回	□ 週2～3回	□ 週2～3回	□ 週2～3回	□ 週2～3回	□ 週2～3回
□ 週1回	□ 週1回	□ 週1回	□ 週1回	□ 週1回	□ 週1回	□ 週1回
□ 週1回未満	□ 週1回未満	□ 週1回未満	□ 週1回未満	□ 週1回未満	□ 週1回未満	□ 週1回未満
□ 食べなかった	□ 食べなかった	□ 食べなかった	□ 食べなかった	□ 食べなかった	□ 食べなかった	□ 食べなかった

		麺類				飲み物
マヨネーズ・ドレッシング	パン（おかずパン・菓子パンも含む）	そば	うどん・ひやむぎ・そうめん	らーめん・インスタントらーめん	スパゲッティ・マカロニなど	緑茶
						□ 毎日4杯以上
□ 毎日2回以上	□ 毎日2回以上	□ 毎日2回以上	□ 毎日2回以上	□ 毎日2回以上	□ 毎日2回以上	□ 毎日2～3杯
□ 毎日1回	□ 毎日1回	□ 毎日1回	□ 毎日1回	□ 毎日1回	□ 毎日1回	□ 毎日1杯
□ 週4～6回	□ 週4～6回	□ 週4～6回	□ 週4～6回	□ 週4～6回	□ 週4～6回	□ 週4～6杯
□ 週2～3回	□ 週2～3回	□ 週2～3回	□ 週2～3回	□ 週2～3回	□ 週2～3回	□ 週2～3杯
□ 週1回	□ 週1回	□ 週1回	□ 週1回	□ 週1回	□ 週1回	□ 週1杯
□ 週1回未満	□ 週1回未満	□ 週1回未満	□ 週1回未満	□ 週1回未満	□ 週1回未満	□ 週1杯未満
□ 食べなかった	□ 食べなかった	□ 食べなかった	□ 食べなかった	□ 食べなかった	□ 食べなかった	□ 飲まなかった

米、パン、麺、コーンフレーク等↓　1日に食べた合計↓

飲み物					「平均的な1日」に食べた“ごはん”と“みそ汁”	
紅茶・ウーロン茶（中国茶）	コーヒー	コーラ・ジュース（スポーツドリンクも含む）	100%果物ジュース 100%野菜ジュース	「主食のある朝食」を食べた頻度	ごはん	みそ汁
□ 毎日4杯以上	□ 毎日4杯以上	□ 毎日4杯以上	□ 毎日4杯以上	□ 毎朝	□ 8杯以上	□ 8杯以上
□ 毎日2～3杯	□ 毎日2～3杯	□ 毎日2～3杯	□ 毎日2～3杯	□ 週に6回	□ 6～7杯	□ 6～7杯
□ 毎日1杯	□ 毎日1杯	□ 毎日1杯	□ 毎日1杯	□ 週に5回	□ 5杯	□ 5杯
□ 週4～6杯	□ 週4～6杯	□ 週4～6杯	□ 週4～6杯	□ 週に4回	□ 4杯	□ 4杯
□ 週2～3杯	□ 週2～3杯	□ 週2～3杯	□ 週2～3杯	□ 週に3回	□ 3杯	□ 3杯
□ 週1杯	□ 週1杯	□ 週1杯	□ 週1杯	□ 週に2回	□ 2杯	□ 2杯
□ 週1杯未満	□ 週1杯未満	□ 週1杯未満	□ 週1杯未満	□ 週に1回	□ 1杯	□ 1杯
□ 飲まなかった	□ 飲まなかった	□ 飲まなかった	□ 飲まなかった	□ 週に1回未満	□ 1杯未満	□ 1杯未満
				□ 食べなかった	□ 食べなかった	□ 食べなかった

コーヒー・紅茶には砂糖を入れますか　□ いつも　□ ときどき　□ いいえ

玄米・胚芽米を食べたり、ごはんに麦や雑穀を混ぜて食べることはありますか？
□ いつも
□ ときどき
□ まれに
□ いいえ

お酒（薬用酒は含めません）

頻度	日本酒	ビール（大瓶で）	焼酎・酎ハイ・泡盛（焼酎・泡盛水割りで）	ウィスキー類（ダブルで）	ワイン（ワイングラスで）
	→1回に飲んだ典型的なお酒の種類の組み合わせとその量				
□ 毎日					
□ 週に6回					
□ 週に5回	□ 4合以上	□ 4本以上	□ 4杯以上	□ 4杯以上	□ 4杯以上
□ 週に4回	□ 3合	□ 3本	□ 3杯	□ 3杯	□ 3杯
□ 週に3回	□ 2合	□ 2本	□ 2杯	□ 2杯	□ 2杯
□ 週に2回	□ 1合	□ 1本	□ 1杯	□ 1杯	□ 1杯
□ 週に1回	□ 0.5合	□ 0.5本	□ 0.5杯	□ 0.5杯	□ 0.5杯
□ 週に1回未満	□ 0.5合未満	□ 0.5本未満	□ 0.5杯未満	□ 0.5杯未満	□ 0.5杯未満
□ 飲まなかった	□ 飲まなかった	□ 飲まなかった	□ 飲まなかった	□ 飲まなかった	□ 飲まなかった

→「飲まなかった」場合には、お酒の種類別の質問に答える必要はありません。

次のページにもお答えください。

あなたは、この1か月のあいだ、以下の食べ物をどのくらいの頻度で食べていましたか？もっともあてはまる回答をひとつ選んで、∨を記入してください。

肉を使った料理（ハム・ソーセージなどの肉加工品も含む）				
焼肉・ステーキ・グリル	ハンバーグ・カレー・ミートソースなど洋風の料理	揚げ物・てんぷら（定食一人前程度の量）	炒め物	和風の煮物・鍋物・どんぶり物・汁物・みそ汁
□ 毎日2回以上	□ 毎日2回以上	□ 毎日2回以上	□ 毎日2回以上	□ 毎日2回以上
□ 毎日1回	□ 毎日1回	□ 毎日1回	□ 毎日1回	□ 毎日1回
□ 週4～6回	□ 週4～6回	□ 週4～6回	□ 週4～6回	□ 週4～6回
□ 週2～3回	□ 週2～3回	□ 週2～3回	□ 週2～3回	□ 週2～3回
□ 週1回	□ 週1回	□ 週1回	□ 週1回	□ 週1回
□ 週1回未満	□ 週1回未満	□ 週1回未満	□ 週1回未満	□ 週1回未満
□ 食べなかった	□ 食べなかった	□ 食べなかった	□ 食べなかった	□ 食べなかった

もっともあてはまる回答をひとつ選んで、∨を記入してください。

お肉（牛肉や豚肉）の脂身は
□ 好んで食べていた
□ やや好んで食べていた
□ 好きでも嫌いでもない
□ あまり食べなかった
□ ほとんど食べなかった

魚を使った料理（いか・たこ・えび・貝も含む）			
さしみ・すし（定食一人前程度の量）	焼き魚	煮魚・鍋物・汁物・みそ汁	てんぷら・揚げ魚（定食一人前程度の量）
□ 毎日2回以上	□ 毎日2回以上	□ 毎日2回以上	□ 毎日2回以上
□ 毎日1回	□ 毎日1回	□ 毎日1回	□ 毎日1回
□ 週4～6回	□ 週4～6回	□ 週4～6回	□ 週4～6回
□ 週2～3回	□ 週2～3回	□ 週2～3回	□ 週2～3回
□ 週1回	□ 週1回	□ 週1回	□ 週1回
□ 週1回未満	□ 週1回未満	□ 週1回未満	□ 週1回未満
□ 食べなかった	□ 食べなかった	□ 食べなかった	□ 食べなかった

麺類のスープ・汁を飲む量は	家庭での味付けは外食と比べて
□ ほとんど全部	□ 薄口
□ 8割くらい	□ 少し薄口
□ 4～6割	□ 同じくらい
□ 2割くらい	□ 少し濃い口
□ ほとんど飲まなかった	□ 濃い口

食事のときに使うしょうゆ・ソース		外食の定食1人前と、自分が普段食べている量を比べると		食べる速さは
頻度は	量は	おかずの量は	ごはんの量は	
□ 必ず使う	□ かなり多め	□ 家のほうがかなり多い	□ 家のほうがかなり多い	□ かなり速い
□ よく使う	□ やや多め	□ 家のほうが少し多い	□ 家のほうが少し多い	□ やや速い
□ ときどき使う	□ ふつう	□ ほぼ同じくらい	□ ほぼ同じくらい	□ ふつう
□ ほとんど使わない	□ やや少なめ	□ 外食のほうが少し多い	□ 外食のほうが少し多い	□ やや遅い
□ まったく使わない	□ かなり少なめ	□ 外食のほうがかなり多い	□ 外食のほうがかなり多い	□ かなり遅い

季節によって食べ方が大きくちがう食べ物

この1年間でもっともよく食べた季節を思い出して、その頃の食べ方についてお答えください。

みかんなどの柑橘(かんきつ)類	かき（柿）	いちご
□ 毎日2回以上	□ 毎日2回以上	□ 毎日2回以上
□ 毎日1回	□ 毎日1回	□ 毎日1回
□ 週4～6回	□ 週4～6回	□ 週4～6回
□ 週2～3回	□ 週2～3回	□ 週2～3回
□ 週1回	□ 週1回	□ 週1回
□ 週1回未満	□ 週1回未満	□ 週1回未満
□ 食べなかった	□ 食べなかった	□ 食べなかった

最後にお答えください。

もっともあてはまる回答をひとつ選んで、∨を記入してください。

この1か月間に栄養補助食品を使いましたか	最近、食事習慣を意識的に変えましたか	現在、医師、栄養士、その他専門家の指導のもとで、食事のコントロールをしていますか	この質問票におもに答えたひとは
□ 毎日2回以上			
□ 毎日1回			
□ 週4～6回	はい	□ はい	□ 本人
□ 週2～3回	□ 3年以上前	□ いいえ	□ 妻
□ 週1回	□ 1～2年前		□ 娘
□ 週1回未満	□ 1年前以内		□ その他
□ 使わなかった	□ いいえ		

消化器症状に関する質問票（出雲スケール）

あなたの過去１週間の状況について質問します。それぞれの質問について、１番よくあてはまるもの１つにチェックをつけてください。

胃酸の逆流のために困ったことがありましたか？（胃酸の逆流とは、少量の苦い水が胃からのどにあがってくる感じをさします）	☐ 全く困らなかった ☐ あまり困らなかった ☐ 少し困った<br>☐ 困った ☐ かなり困った ☐ 我慢できないくらい困った
前胸部に熱く焼けるような感じがして困ったことがありましたか？	☐ 全く困らなかった ☐ あまり困らなかった ☐ 少し困った<br>☐ 困った ☐ かなり困った ☐ 我慢できないくらい困った
のどの違和感で困ったことがありましたか？（のどの違和感とはひとによって異なりますが、何かが詰まっている感じや、ヒリヒリした感じ、何かに摘まれている感じをさします）	☐ 全く困らなかった ☐ あまり困らなかった ☐ 少し困った<br>☐ 困った ☐ かなり困った ☐ 我慢できないくらい困った
胃が痛くて困ったことがありましたか？（空腹時の痛みは除く）	☐ 全く困らなかった ☐ あまり困らなかった ☐ 少し困った<br>☐ 困った ☐ かなり困った ☐ 我慢できないくらい困った
空腹時に胃が痛くて困ったことがありましたか？	☐ 全く困らなかった ☐ あまり困らなかった ☐ 少し困った<br>☐ 困った ☐ かなり困った ☐ 我慢できないくらい困った
みぞおちの辺り（おへそと胸の問）が焼けるような熱い感じで困ったことがありましたか？	☐ 全く困らなかった ☐ あまり困らなかった ☐ 少し困った<br>☐ 困った ☐ かなり困った ☐ 我慢できないくらい困った
食事をするとすぐにおなかがいっぱいになって困ったことがありましたか？	☐ 全く困らなかった ☐ あまり困らなかった ☐ 少し困った<br>☐ 困った ☐ かなり困った ☐ 我慢できないくらい困った
食後に胃の中にいつまでも食べ物がとどまっているような重苦しく、ムカムカした感じがあって困ったことがありましたか？	☐ 全く困らなかった ☐ あまり困らなかった ☐ 少し困った<br>☐ 困った ☐ かなり困った ☐ 我慢できないくらい困った
胃の彫満感のために困ったことがありましたか？（胃の膨満感とは.胃にガスがたまっておなかが張っている感じをさします）	☐ 全く困らなかった ☐ あまり困らなかった ☐ 少し困った<br>☐ 困った ☐ かなり困った ☐ 我慢できないくらい困った
完全に便を出しきれていない感じ（残便感）で困ったことがありましたか？	☐ 全く困らなかった ☐ あまり困らなかった ☐ 少し困った<br>☐ 困った ☐ かなり困った ☐ 我慢できないくらい困った
何日も続く便秘あるいは硬い便で困ったことがありましたか？	☐ 全く困らなかった ☐ あまり困らなかった ☐ 少し困った<br>☐ 困った ☐ かなり困った ☐ 我慢できないくらい困った
強いストレスを感じた時におこる便秘で困ったことがありましたか？	☐ 全く困らなかった ☐ あまり困らなかった ☐ 少し困った<br>☐ 困った ☐ かなり困った ☐ 我慢できないくらい困った
急な便意でトイレに駆け込みたくなるような感じ（便意切迫感） で困ったことがありましたか？（便意切迫感とは、便が出そうになる状態をさします）	☐ 全く困らなかった ☐ あまり困らなかった ☐ 少し困った<br>☐ 困った ☐ かなり困った ☐ 我慢できないくらい困った
下痢あるいは軟らかい便で困ったことがありましたか？	☐ 全く困らなかった ☐ あまり困らなかった ☐ 少し困った<br>☐ 困った ☐ かなり困った ☐ 我慢できないくらい困った
強いストレスを感じた時に起こる下痢で困ったことかありましたか？	☐ 全く困らなかった ☐ あまり困らなかった ☐ 少し困った<br>☐ 困った ☐ かなり困った ☐ 我慢できないくらい困った

回答にあたっては以下の点にご注意ください。
◆**強い身体活動**とは、身体的にきついと感じるような、かなり呼吸が乱れるような活動を意味します。
◆**中等度の身体活動**とは、身体的にやや負荷がかかり、少し息がかすむような活動を意味します。

第1部：仕事中の身体活動に関する質問
まず最初に、仕事中の身体活動についてお尋ねします。ここで言う仕事とは、有給の仕事、自営業、農作業、ボランティア活動、学業、無給の仕事などのことです。家庭で行っている活動（家事、庭仕事、自宅の手入れ、家族の介護など）は含めないで下さい。これについては第3部で行っている活動（家事、庭仕事、自宅の手入れ、家族の介護など）でお尋ねします。

質問1a	現在、有給、無給を問わず何か仕事をお持ちですか？	□ はい<br>□ いいえ（→第2部：移動の身体活動に関する質問へ）
質問1a1	上記で、はいの場合、ご職業をご記入ください。	
質問1a2	交代制勤務はありますか	□ はい　□ いいえ
質問1a3	夜勤はありますか	□ はい　□ いいえ

これから、平均的な1週間における、仕事中の身体活動についてお尋ねします。ここでは通勤は含めないで下さい。
1回につき少なくとも10分間以上続けて行なう仕事中の身体活動についてのみ考えて、お答え下さい。

質問1b	平均的な1週間では、仕事中に強い身体活動（重い荷物の運搬作業、肉体労働など）を行なう日は何日ありますか？	□ 週 ____日　□ ない（→質問1dへ）
質問1c	仕事中に強い身体活動を行なう日には、通常、1日合計でどのくらいの時間そのような作業を行ないますか？	1日 ____時間 ____分
質問1d	平均的な1週間では、仕事中に中等度身体活動（軽い荷物の運搬作業など）を行なう日は何日ありますか？	□ 週 ____日　□ ない（→質問1fへ）
質問1e	仕事中に中等度の身体活動を行なう日には、通常、1日合計でどのくらいの時間そのような作業を行ないますか？	1日 ____時間 ____分
質問1f	平均的な1週間では、仕事中に少なくとも10分以上続けて歩く日は何日ありますか？通勤時の歩行は含めないで考えて下さい。	□ 週 ____日<br>□ ない（→第2部：移動の身体活動に関する質問へ）
質問1g	仕事中に少なくとも10分以上続けて歩く日には、通常、1日合計でどのくらいの時間歩きますか？	1日 ____時間 ____分

第2部：移動の身体活動に関する質問
ここでは、さまざまな場所へ移動するとき(通勤、買い物、映画を見に行くときなど)にどのような方法で移動しているのかについてお尋ねします。

質問2a	平均的な1週間では、電車、バス、車、オートバイなどの乗り物（自転車は含まない）を利用する日は何日ありますか？	□ 週 ____日　□ ない（→質問2cへ）
質問2b	乗り物を利用する日には、電車、バス、車、オートバイなどの乗り物（自転車は含まない）に、通常、1日合計でどのくらいの時間乗りますか？	1日 ____時間 ____分

次に、自転車と歩行による移動（通勤、お使いなど）について考えて下さい。

質問2c	平均的な1週間では、移動のために少なくとも10分以上続けて自転車に乗る日は何日ありますか？	□ 週 ____日　□ ない（→質問2eへ）
質問2d	移動のために自転車に乗る日には、通常、1日合計でどのくらいの時間自転車に乗りますか？	1日 ____時間 ____分
質問2e	平均的な1週間では、移動のために少なくとも10分以上続けて歩く日は何日ありますか？	□ 週 ____日<br>□ ない<br>（→第3部：家事など自宅での身体活動に関する質問へ）
質問2f	移動のために歩く日には、通常、1日合計で何分くらい歩きますか？	1日 ____時間 ____分

第3部：家事、家の手入れ、家族の介護など、自宅での身体活動に関する質問

ここでは、自宅での身体活動（家事、庭仕事、家の手入れ、家族の介護など）についてお尋ねします。
ここでも、1回につき少なくとも10分間以上続けて行なう身体活動についてのみ考えて、お答え下さい。

質問3a	平均的な1週間では、庭で強い身体活動（重い荷物を持ち運んだり、穴を掘ったり、雪かきをしたり、かなり呼吸が乱れるような作業）を行なう日は何日ありますか？	☐ 週 ____日　☐ 行わない（→質問3cへ）
質問3b	庭で強い身体活動を行なう日には、通常、1日合計でどのくらいの時間そのような作業を行ないますか？	1日 ____時間 ____分
質問3c	平均的な1週間では、庭で中等度の身体活動（軽い荷物を持ち運ぶことなど、少し息のはずむような作業）を行なう日は何日ありますか？	☐ 週 ____日　☐ 行わない（→質問3eへ）
質問3d	庭で中等度の身体活動を行なう日には、通常、1日合計してどのくらいの時間そのような作業を行ないますか？	1日 ____時間 ____分
質問3e	平均的な1週間では、家の中で中等度の身体活動（軽い荷物を持ち運ぶこと、床の拭き掃除、力を使う老人の介護、子供と動き回って遊ぶことなど少し息のはずむような活動）を行なう日は何日ありますか？	☐ 週 ____日<br>☐ 行わない（→第4部：レクリエーション、運動、レジャーなどでの身体活動に関する質問へ）
質問3f	家の中で中等度の身体活動を行なう日には、通常、1日合計してどのくらいの時間そのような活動を行ないますか？	1日 ____時間 ____分

第4部：レクリエーション、運動、レジャーなどでの身体活動に関する質問
ここでは、純粋にレクリエーション、スポーツ、運動、レジャーとして行っている身体活動に関してお尋ねします。ここでも、1回につき少なくとも10分間以上続けて行なう身体活動についてのみお答え下さい。なお、ここまでの質問でお答えいただいた身体活動は含めないで下さい。

質問4a	これまでお答えいただいた歩行（仕事中や移動での歩行）については含めないでお答え下さい。平均的な1週間では、余暇時間に散歩やウォーキングを10分以上続けて行なう日は何日ありますか？	☐ 週 ____日　☐ ない（→質問4cへ）
質問4b	余暇として散歩やウォーキングをする日には、通常、1日合計してどのくらいの時間歩きますか？	1日 ____時間 ____分
質問4c	平均的な1週間では、余暇として強身体活動（ジョギング、速く泳ぐ、激しいエアロビクス、バスケットボール、登山など）を行なう日は何日ありますか？	☐ 週 ____日　☐ ない（→質問4eへ）
質問4d	余暇として強い身体活動を行なう日には、通常、1日合計してどのくらいの時間、そのような活動を行ないますか？	1日 ____時間 ____分
質問4e	平均的な1週間では、余暇として中等度の身体活動（ゆっくり泳ぐこと、テニスのダブルス、野球、平地でのハイキングなど）を行なう日は何日ありますか？	☐ 週 ____日<br>☐ ない（→第5部：非活動的な時間に関する質問へ）
質問4f	余暇として中等度の身体活動を行なう日には、通常、1日合計してどのくらいの時間、そのような活動を行ないますか？	1日 ____時間 ____分

第5部：非活動的な時間に関する質問

最後に、毎日座ったり寝転んだりして過ごしている時間（仕事中、自宅で、勉強中、余暇時間など）についてお尋ねします。すなわち、机に向かったり、友人とおしゃべりをしたり、読書をしたり、座ったり、寝転んでテレビを見たり、といった非活動的な時間全てを含みます。睡眠時間は含めないで下さい。また、車の運転や、電車やバスに乗っている時間については、すでにお尋ねしていますので、ここでは含めないで下さい。

質問5a	平日には、通常、1日合計でどのくらいの時間、座ったり寝転んだりして過ごしますか？	1日 ____時間 ____分
質問5b	休日には、通常、1日合計でどのくらいの時間、座ったり寝転んだりして過ごしますか？	1日 ____時間 ____分

過去1ヵ月間に、少なくとも週３回以上経験したものを選んでください。

1	寝床についてから実際に寝るまで、<br>時間がかかりましたか？	☐ いつもより寝つきは良い<br>☐ いつもより少し時間がかかった<br>☐ いつもよりかなり時間がかかった<br>☐ いつもより非常に時間がかかった、あるいは全く眠れなかった
2	夜間、睡眠の途中で目が覚めましたか？	☐ 問題になるほどのことはなかった<br>☐ 少し困ることがある<br>☐ かなり困っている<br>☐ 深刻な状態、あるいは全く眠れなかった
3	希望する起床時間より早く目覚めて、<br>それ以降、眠れないことはありましたか？	☐ そのようなことはなかった<br>☐ 少し早かった<br>☐ かなり早かった<br>☐ 非常に早かった、あるいは全く眠れなかった
4	夜の眠りや昼寝も合わせて、<br>睡眠時間は足りてましたか？	☐ 十分である<br>☐ 少し足りない<br>☐ かなり足りない<br>☐ 全く足りない、あるいは全く眠れなかった
5	全体的な睡眠の質について、<br>どう感じていますか？	☐ 満足している<br>☐ 少し不満である<br>☐ かなり不満である<br>☐ 非常に不満である、あるいは全く眠れなかった
6	日中の気分はいかがでしたか？	☐ いつもどおり<br>☐ 少し滅入った<br>☐ かなり滅入った<br>☐ 非常に滅入った
7	日中の身体的および精神的な活動の状態は、<br>いかがでしたか？	☐ いつもどおり<br>☐ 少し低下した<br>☐ かなり低下した<br>☐ 非常に低下した
8	日中の眠気はありましたか？	☐ 全くなかった<br>☐ 少しあった<br>☐ かなりあった<br>☐ 激しかった
9	毎日だいたい何時に起きますか？	☐ 午前　☐ 午後　________時　__________分
10	毎日だいたい何時に寝ますか？	☐ 午前　☐ 午後　________時　__________分
11	だいたい何時間寝ていますか？	________時間　__________分
12	週に何日程度、夜寝ている間にトイレのために起きますか？	週に　__________日
	その場合、夜寝ている間に１日何回トイレに行きますか？	夜１日に　__________回

問1	あなたの体調が最高と思われる生活リズムだけを考えて下さい。そのうえで、1日のスケジュールを本当に思い通りに組むことができるとしたら、あなたは何時に起きますか。	☐ 午前 ☐ 午後<br>________時 __________分
問2	あなたの体調が最高と思われる生活リズムだけを考えて下さい。そのうえで、夜のすごし方を本当に思い通りに計画できるとしたら、あなたは何時に寝ますか。	☐ 午前 ☐ 午後<br>________時 __________分
問3	朝、ある特定の時刻に起きなければならないとき、どの程度目覚し時計に頼りますか。	☐ まったく頼らない ☐ あまり頼らない ☐ わりに頼る<br>☐ たいへん頼る
問4	ふだんあなたは、朝、目が覚めてから容易に起きることができますか。	☐ まったく容易でない ☐ あまり容易でない ☐ わりに容易である<br>☐ たいへん容易である
問5	ふだん、起床後30分間の目覚めぐあいは、どの程度ですか。	☐ まったく目覚めていない ☐ あまり目覚めていない<br>☐ わりに目覚めている ☐ たいへん目覚めている
問6	ふだん、起床後30分間の食欲は、どの程度ですか。	☐ まったく食欲がない ☐ あまり食欲がない ☐ わりに食欲がある<br>☐ たいへん食欲がある
問7	ふだん、起床後30分間のけだるさは、どの程度ですか。	☐ たいへんけだるい ☐ どちらかといえばけだるい<br>☐ どちらかといえばそう快である ☐ たいへんそう快である
問8	次の日、まったく予定がないとすれば、あなたは寝る時刻をいつもに比べてどうしますか。	☐ 遅くすることはほとんどない（まったくない）<br>☐ 遅くしても1時間以内<br>☐ 1-2時間遅くする ☐ 2時間以上遅する
問9	何か運動をしようと思いたちました。友人が「それならば、週2回1時間ずつで、時刻は午前7時から午前8時までが一番いい」と助言してくれました。あなたの体調が最高と思われる生活リズムだけを考えると、それをどの程度やりぬけると思いますか。	☐ 完全に実行できるだろうと思う<br>☐ わりに実行できるだろうと思う<br>☐ 実行するのは難しいだろうと思う<br>☐ 実行するのはたいへん難しいだろうと思う
問10	あなたは、夜、何時になると疲れを感じ、眠くなりますか。	☐ 午前 ☐ 午後 ________時 __________分
問11	精神的にたいへん疲れるうえ、2時間もかかるとわかっているテストを受けて、最高の成績をあげたいとします。1日のスケジュールを本当に思い通りに組むことができ、あなたの体調が最高と思われる生活リズムだけを考えると、次のうちどの時間帯を選びますか。	☐ 午前8時～午前10時<br>☐ 午前11時～午後1時<br>☐ 午後3時～午後5時<br>☐ 午後7時～午後9時

問12	午後11時に寝るとすれば、あなたは、そのときどの程度疲れていると思いますか。	☐ まったく疲れていないと思う　☐ あまり疲れていないと思う<br>☐ わりに疲れていると思う　☐ たいへん疲れていると思う
問13	ある理由で寝るのがいつもより何時間か遅くなったが、翌朝は特定の時刻に起きる必要がない場合、あなたは次のどれにあてはまりますか。	☐ いつもの時刻に目覚め、それ以上眠らないだろう<br>☐ いつもの時刻に目覚めるが、その後うとうとするだろう<br>☐ いつもの時刻に目覚めるが、また眠るだろう<br>☐ いつもの時刻より遅くまで目覚めないだろう
問14	ある夜、夜警のため午前4時から午前6時まで起きていなければならないが、次の日はまったく予定がないとします。あなたは次のどれにもっともよくあてはまりますか。	☐ 夜警が終わるまで寝ないだろう<br>☐ 夜警前に仮眠をとり、夜警後に眠るだろう<br>☐ 夜警前に十分眠り、夜警後に仮眠をとるだろう<br>☐ 夜警前にできる限り眠るだろう
問15	きつい肉体作業を2時間しなければなりません。1日のスケジュールを本当に思い通りに組むことができ、あなたの体調が最高と思われる生活リズムだけを考えると、次のうちのどの時間帯を選びますか。	☐ 午前8時～午前10時<br>☐ 午前11時～午後1時<br>☐ 午後3時～午後5時<br>☐ 午後7時～午後9時
問16	きつい運動をしようと思いたちました。友人が「それならば、週2回1時間ずつで、時刻は午後10時から午後11時までが一番いい」と助言してくれました。あなたの体調が最高と思われる生活リズムを考えると、それをどの程度やりぬけると思いますか。	☐ 完全に実行できるだろうと思う<br>☐ わりに実行できるだろうと思う<br>☐ 実行するのは難しいだろうと思う<br>☐ 実行するのはたいへん難しいだろうと思う
問17	仕事をする時間帯を、あなた自身で選ぶことができるとします。おもしろいうえ、できばえに応じて報酬がある仕事を5時間連続して（休憩を含む）行うとき、何時開始がいいですか。	開始時間　☐　午前　☐　午後　________時
問18	1日のどの時間帯に体調が最高であると思いますか。	☐　午前　☐　午後　________時ごろ
問19	「朝型」か「夜型」かと尋ねられたら、あなたは次のうちどれにあてはまりますか。	☐ 明らかに「朝型」　☐ 「夜型」というよりむしろ「朝型」<br>☐ 「朝型」というよりむしろ「夜型」　☐ 明らかに「夜型」

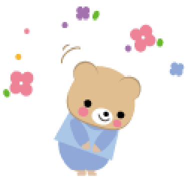

ご記入ありがとうございました。

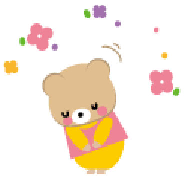

Supplement: Supplementary file 1 — Additional file 1:. [file 12882_2021_2271_MOESM1_ESM.pdf]
