# Supplementary material for: Low circulating arachidonic acid is associated with macroalbuminuria in diabetic patients: a cross-sectional examination of the KAMOGAWA-DM cohort study
Source: BMC Nephrol. 2021 Feb 23;22:68. doi: 10.1186/s12882-021-02271-8 (PMC7903748; doi:10.1186/s12882-021-02271-8)
Supplement: Supplementary file 2 — Additional file 2:. [file 12882_2021_2271_MOESM2_ESM.docx]

**Page 1**

Informed consent

**Page 2**

Name of institution

Study ID

Entry date

Date of birth

Gender

Postal code

How old were you when you were told you had diabetes?

What is your current height and weight?

What was your weight at age 20 and what was your maximum weight and age at that time?

- About your family

Do you have any blood relatives with diabetes?

- About your lifestyle

Do you smoke?

If you smoke, how long do you smoke?

If you smoked, how long did you smoke?

Do you drink?

Does it turn red when you drink?

Do you have cohabitants?

Tell me about your marriage.

What was your last education?

- About your diet

What is the approximate time of the meal?

Do you eat breakfast every day?

Do you eat out for dinner?

Do you have a late-night snack?

Do you snack?

What time is the late-night snack /snack time?

Which is the largest amount of breakfast, lunch or dinner?

**Page3-5**

- BDHQ

This section is detailed in the following paper.

Kobayashi S, Murakami K, Sasaki S, Okubo H, Hirota N, Notsu A, et al. Comparison of relative validity of food group intakes estimated by comprehensive and brief-type self-administered diet history questionnaires against 16 d dietary records in Japanese adults. Public Health Nutr 2011;14:1200–11. <https://doi.org/10.1017/S1368980011000504>.

**Page 6**

This section is detailed in the following paper.

Furuta K, Ishihara S, Sato S, et al. Nihon Shokakibyo Gakkai Zasshi. 2009;106(10):1478‐1487.

Page 7

- Questions about physical activity at work

Do you currently have any jobs, paid or unpaid?

If yes, please indicate your occupation.

Do you offer shift work?

Do you offer a night shift?

How many days in an average week do you engage in intense physical activity (heavy lifting, physical labor, etc.) at work?

On days when you engage in intense physical activity at work, how long do you typically spend doing this work?

In an average week, how many days of moderate physical activity (e.g., light lifting) do you engage in at work?

On days of moderate physical activity at work, how long do you typically spend doing this work for a total of one day?

In an average week, how many days do you walk for at least 10 minutes in a row at work? Please don't include walking to work and think about it.

On days when you walk for at least 10 minutes in a row at work, how long do you typically walk for a total of one day?

- Questions about physical activity of mobility

In an average week, how many days do you use trains, buses, cars, motorcycles, and other vehicles (not including bicycles) in an average week?

How long do you typically ride trains, buses, cars, motorcycles, and other vehicles (not including bicycles) for a total of how long in a day on a transit day?

In an average week, how many days do you ride your bicycle for at least 10 minutes?

On days when you ride your bike to get around, how long do you typically ride your bicycle for a total of one day?

In an average week, how many days do you have to walk for at least 10 minutes?

On days when you walk to get around, how many minutes do you typically walk for a total of one day?

**Page 8**

This section is detailed in the following paper.

Murase N, Katsumura T, Ueda C, Inoue S, Shimomitsu T. Validity and reliability of Japanese version of International Physical Activity Questionnaire. Journal of Health and Welfare Statistics. 2002;49(11):1-9.

**Page 9**

This section is detailed in the following paper.

Soldatos CR, Dikeos DG, Paparrigopoulos TJ. Athens Insomnia Scale: validation of an instrument based on ICD-10 criteria. J Psychosom Res. 2000;48(6):555‐560.

**Page 10-11**

This section is detailed in the following paper.

Ishihara K, Miyashita A, Inugami M, Fukuda K, Yamazaki K, Miyata Y. Shinrigaku Kenkyu. 1986;57(2):87‐91. doi:10.4992/jjpsy.57.87
